# Curcumin-Induced Apoptotic Cell Death in Human Glioma Cells Is Enhanced by Clusterin Deficiency

**DOI:** 10.3390/pharmaceutics17060679

**Published:** 2025-05-22

**Authors:** Pinky Sultana, Jiri Novotny

**Affiliations:** Department of Physiology, Faculty of Science, Charles University, 128 00 Prague, Czech Republic; pinky.sultana@natur.cuni.cz

**Keywords:** apoptosis, cellular senescence, clusterin, curcumin, glioma, oxidative stress

## Abstract

**Background/Objectives:** Glioblastoma is an aggressive brain tumor with limited treatment options and significant resistance to conventional therapies. **Methods:** In this study, we explored the effects of combining curcumin treatment with clusterin inhibition on cell death in glioma cells. **Results:** We observed that the combination of clusterin silencing and curcumin treatment induces cell death. This combination therapy significantly elevated reactive oxygen species (ROS), triggering oxidative stress, which acted as a key upstream mediator of apoptosis. Elevated ROS levels were found to be associated with caspase activation, suggesting apoptosis as the primary mode of cell death. Furthermore, autophagy was induced as a complementary mechanism, with upregulation of LC3B contributing to the enhanced cytotoxic effects. **Conclusions:** The synergy between clusterin knockdown-induced senescence and curcumin’s pro-apoptotic and pro-autophagic effects highlights a potential novel therapeutic strategy for gliomas. These findings underscore the potential of this combination therapy in overcoming glioma resistance and improving treatment outcomes through the dual induction of oxidative stress and cell death pathways.

## 1. Introduction

Astrocytes, a common type of glial cell, may begin to grow abnormally under certain situations, which can lead to a glioma or its severe form, glioblastoma, a highly aggressive type of central nervous system (CNS) tumor. Gliomas and glioblastomas (GBMs) refer to gliomas of astrocytic origin [1]. Gliomas are difficult to treat because the tumors are interspersed with healthy tissue in the brain. These tumors pose significant challenges in treatment due to their resistance to standard therapies. Consequently, patients afflicted with gliomas typically face a poor prognosis, with a survival time ranging from 12 to 14 months [2,3]. Patients diagnosed with glioma usually undergo the standard treatment regimen, which includes surgical intervention followed by radiotherapy, in addition to concurrent and subsequent chemotherapy, often utilizing the alkylating agent temozolomide (TMZ) [4]. However, the prognosis remains poor due to the inability of cytotoxic drugs to cross the blood–brain barrier [5]. Even with post-surgical TMZ chemotherapy, a majority of patients experience tumor recurrence at the primary site, indicating the development of TMZ resistance in the glioma [6]. This TMZ resistance is mainly due to DNA damage repair by the enzyme O6-methylguanine-DNA methyltransferase [7]. Therefore, novel approaches to suppress uncontrolled glioma cell growth may be crucial for achieving more successful glioma treatment.

Clusterin (CLU), also known as apolipoprotein J, is a glycoprotein involved in many cellular functions, including cell adhesion, membrane transport recycling, immune response modulation, and the regulation of cell survival and apoptosis [8]. CLU exhibits heightened expression across various cancers, including prostate, breast, and lung cancers. Extensive research suggests that CLU’s diverse isoforms contribute to several physiological processes, such as tumorigenesis, programmed cell death, tumor metastasis, invasion, and epithelial–mesenchymal transition [9]. Notably, CLU has been linked to protecting cells from apoptosis induction. Elevated CLU levels confer resistance to TNF-α-triggered cell death, and its overexpression inhibits cellular demise [10], while its depletion reduces cell growth and increases sensitization to genotoxic and oxidative stress, including apoptosis in cancer cells [11]. The expression of CLU could be suppressed by custirsen (OGX-011), an advanced antisense oligonucleotide engineered to inhibit mRNA encoding the sCLU protein. It has been extensively studied in prostate cancer and implemented in clinical trials [12]. However, it has not shown significantly higher survival rates in phase III clinical trials, either alone or in combination with anticancer drugs like docetaxel or prednisone [12]. A recent study demonstrated that CLU suppression induces senescence and reduces proliferation in pancreatic cancer cell lines; however, apoptosis markers showed no significant changes in CLU-silenced cells, leaving the underlying mechanism unclear [13]. Interestingly, CLU deficiency caused senescence and mitochondrial dysfunction in the CCF-STTG1 glioma cell line, known for its high-grade and aggressive characteristics, as well as in immortalized human astrocytes [14]. As recently explored in a comprehensive review, the potential of CLU inhibition to induce senescence, when combined with natural senolytics, could be used to accelerate the suppression of cancer cell growth [15].

Curcumin, derived from the root of the turmeric plant, is a yellow-orange pigment and a key ingredient in curry powder, commonly used in Asian cuisine. Rooted in traditional Indian medicine, curcumin has garnered significant scientific attention, particularly in cancer research. Previous studies have highlighted curcumin’s notable antioxidant, anti-inflammatory, and anticancer properties [16]. A study on intervertebral disc cells revealed the senolytic effect of curcumin in downregulating NF-κB and increasing apoptosis in the degenerating cells [17].

In this study, we investigated the effect of curcumin on glioma cell proliferation and explored the potential impact of manipulating CLU expression on curcumin’s efficacy in inducing cell death. In parallel, we examined these effects in SV40-immortalized normal human astrocytes. In addition to assessing cell viability and senescence under different experimental conditions, we analyzed the involvement of apoptotic and autophagic processes, as well as key survival pathways, in the observed phenomena.

## 2. Materials and Methods

### 2.1. Cell Lines and Culture Conditions

The human brain glioma cell line CCF-STTG1 (CCF), obtained from the European Collection of Authenticated Cell Cultures, and SV40-immortalized normal human astrocytes (INHA), obtained from Innoprot, Spain, were cultured in DMEM (Sigma-Aldrich, St. Louis, MO, USA) supplemented with 10% fetal bovine serum, 100 U/mL penicillin, and 100 µg/mL streptomycin (Sigma-Aldrich, St. Louis, MO, USA). Cells were maintained at 37 °C in a humidified atmosphere containing 5% CO_2_. All experiments were performed using cells maintained in culture for a maximum of two months.

### 2.2. siRNA Transfection

Small interfering RNA reverse transfection FCells were transfected with 25 nM CLU siRNA (siCLU, siRNA ID s3156, Thermo Fisher Scientific, Waltham, MA, USA, #4392420) or non-targeting siRNA (siNC, Thermo Fisher Scientific, #4390843) using Lipofectamine RNAiMAX transfection reagent (Invitrogen, Carlsbad, CA, USA), following the manufacturer’s protocol. To induce a senescent phenotype, cells were incubated with the siRNA transfection mixture for 72 h [14].

### 2.3. Cell Proliferation Assay

The colorimetric MTT assay was used to assess cellular metabolic activity as an indicator of cell viability and proliferation. Cells were seeded at a density of 7500 cells per well in a 96-well plate and treated with various concentrations of curcumin (Sigma-Aldrich, St Louis, MI, USA, C1386) for 24 h to determine the optimal concentration for proliferation inhibition.

### 2.4. Curcumin Treatment

CCF and INHA cells were initially treated with varying concentrations of curcumin (ranging from 1 to 25 μM) for 24 h to determine the optimal dose that would result in approximately a 30% reduction in cell proliferation, as assessed by the MTT assay. For all subsequent experiments, cells were treated with 10 μM curcumin for 24 h following 72 h of CLU siRNA transfection-induced senescence, and then subjected to further analyses.

### 2.5. Crystal Violet Assay

The crystal violet assay was used as a screening method to evaluate the impact of CLU downregulation on cell survival and growth inhibition (https://openwetware.org/, accessed on 23 November 2024). CCF and INHA cells were seeded at a density of 250,000 cells in a 6-well plate and reverse transfected with siCLU or siNC. After 72 h, cells were treated with 10 μM curcumin for 24 h, then fixed with methanol at 4 °C for 20 min. Growth arrest was assessed using crystal violet staining (0.5% crystal violet solution in 25% methanol) for 10 min, and images were captured under white light.

### 2.6. Scratch Wound Migration Assay

For the scratch wound assay, 60,000 cells were reverse-transfected and seeded into 12-well plates. After 2 h, a 1 mm pipette tip was used to create a scratch in the monolayer. The cells were then further incubated for 24 to 72 h, followed by treatment with curcumin for 24 h. Images of the scratched monolayers were captured using a phase-contrast microscope at 4× magnification. The scratch border distance was measured using ImageJ (https://imagej.net/ij/download.html, accessed on 11 September 2024), and the cell migration speed was calculated from three independent experiments.

### 2.7. Automated Cell Counting

Cells transfected with CLU siRNA or non-targeting siRNA were cultured for up to 72 h, followed by an additional 24 h with or without curcumin. Cell imaging was performed for up to 12 days using the BioTek Cytation 5 cell imaging multimode reader (Agilent Technologies, Inc., Santa Clara, CA, USA) at 4× magnification. Automated cell counting was conducted using the Gen5 software integrated with the Cytation 5 system. Cell growth quantification was based on data from three independent experiments.

### 2.8. Acridine Orange and Propidium Iodide Staining

Cells transfected with CLU siRNA or non-targeting siRNA were cultured for up to 72 h, followed by 24 h with or without curcumin. A working solution of acridine orange (AO) and propidium iodide (PI) was prepared at a concentration of 1 μg/mL. Cells were immediately stained with this solution for 3–5 min. Fluorescent images were captured using a fluorescent microscope (AIF 5013i-T, Arsenal, Prague, Czechia) and apoptotic and necrotic cells were quantified using the ImageJ software. Green fluorescence indicated viable cells, while orange and red signified early apoptosis and necrosis, respectively.

### 2.9. Assessment of Apoptosis Using Flow Cytometry

Apoptotic cells were identified using Annexin V-FITC and Hoechst 33342 staining (Sigma-Aldrich, St. Louis, MO, USA). Cells were seeded in 6-well plates and treated according to the experimental conditions. Following treatment, they were harvested by trypsinization and washed twice with cold phosphate-buffered saline (PBS). Approximately 1 × 10^5^ cells were resuspended in 100 µL of 1× Annexin V binding buffer. Annexin V-FITC and Hoechst 33342 stains were added to the suspension, mixed gently, and incubated in the dark at room temperature for 15 min. After incubation, 400 µL of binding buffer was added, and samples were analyzed immediately using flow cytometry (BD LSR II flow cytometer, BD Biosciences, San Jose, CA, USA). All experiments were conducted in triplicate.

### 2.10. NADPH Assay

NADPH levels were measured using the NADPH assay kit (ab186031, Abcam, Cambridge, UK). Cells transfected with siCLU or siNC in 6-well plates were incubated for 72 h, followed by treatment with or without curcumin for 24 h. Cells were then lysed using the kit’s lysis buffer, sonicated, and centrifuged at 14,000× *g* for 15 min at 4 °C. NADPH in the supernatant was detected by adding the NADPH probe and absorbance at 460 nm was measured using a BioTek Synergy HT microplate reader (BioTek Instruments, Inc., Winooski, VT, USA).

### 2.11. Measurement of H_2_O_2_ Using a Fluorometric Assay

H_2_O_2_ levels were measured fluorometrically using the Amplex^TM^ Red Hydrogen Peroxide Assay Kit (Cat# A22188, Thermo Fisher Scientific, Waltham, MI, USA), according to the manufacturer’s protocol. Cells were transfected with siCLU or siNC in 6-well plates for 72 h, followed by treatment with or without curcumin for an additional 24 h. H_2_O_2_ accumulation was then measured in the extracellular medium. Fluorescence intensity was detected using a BioTek Synergy HT microplate reader at an excitation wavelength of 530 nm and an emission wavelength of 590 nm, at room temperature.

### 2.12. Nitric Oxide Assay

The Griess reaction was used to measure NO production in astrocytes [18]. Cells were transfected with siCLU or siNC in 6-well plates for 72 h, followed by treatment with or without curcumin for an additional 24 h. Subsequently, 50 μL of cell culture medium were collected and mixed with an equal volume of Griess reagent (0.1% N-1-naphthylethylenediamine dihydrochloride and 1% sulfanilamide in 5% phosphoric acid) in a 96-well plate. The mixture was incubated for 5–10 min at room temperature, and absorbance was measured at 540 nm using BioTek Synergy HT microplate reader.

### 2.13. Determination of Reduced Glutathione

Reduced glutathione (GSH) levels were measured using the Reduced Glutathione Assay Kit (ab239709, Abcam, Cambridge, UK). Cells transfected with siCLU or siNC in 6-well plates were incubated for 72 h, followed by treatment with or without curcumin for an additional 24 h. The cells were then lysed using the lysis buffer provided in the kit and centrifuged at 14,000× *g* for 10 min. GSH in the supernatant reacted with DTNB to produce yellow 2-nitro-5-thiobenzoic acid, whose absorbance was measured using a BioTek Synergy HT microplate reader.

### 2.14. Protein Determination

Protein concentration was determined using the bicinchoninic acid (BCA) assay. Samples were incubated with copper ions and BCA in an alkaline environment at 37 °C for 30 min. Absorbance was then measured at 562 nm using a microplate reader.

### 2.15. Western Blotting Analysis

Western blotting analysis was performed as previously described [14]. Briefly, cells were lysed in RIPA lysis buffer containing a protease inhibitor cocktail (Sigma-Aldrich). Equal amounts of protein from the lysates were mixed with 4× Laemmli buffer and boiled for 2 min prior to loading onto a gel (Bio-Rad, Hercules, CA, USA). Proteins were separated at a constant voltage (200 V) and electrophoretically transferred onto 0.45 μM or 0.2 μM nitrocellulose membranes using the Trans-Blot Turbo system (Bio-Rad). Membranes were blocked with 5% BSA at room temperature for 1 h and incubated at 4 °C overnight with primary antibodies. After three washes with TBS containing 0.1% Tween-20 (wash buffer), membranes were incubated for 1 h at room temperature with horseradish peroxidase-conjugated secondary antibodies (anti-mouse, anti-rabbit, or anti-goat IgG; Santa Cruz Biotechnology, Inc., Dallas, TX, USA). Following three additional washes, membranes were developed using the FEMTO chemiluminescent substrate according to the manufacturer’s instructions (Pierce Biotechnology, Rockford, IL, USA). The resulting blots were scanned and quantitatively analyzed using the ImageJ software. Protein expression levels were normalized to Ponceau S or beta-actin staining and expressed as fold changes relative to the loading control.

### 2.16. Statistical Analysis

All experiments were performed in at least three independent biological replicates. Statistical analyses were conducted using GraphPad Prism software version 8.0 (GraphPad Software, San Diego, CA, USA). Data are expressed as the mean ± SEM (standard error of the mean). Differences between groups were analyzed using one-way ANOVA followed by the Bonferroni post hoc test, with *p*-values less than 0.05 considered statistically significant.

## 3. Results

### 3.1. Curcumin Suppresses Cell Viability and Motility

**Figure 1:**
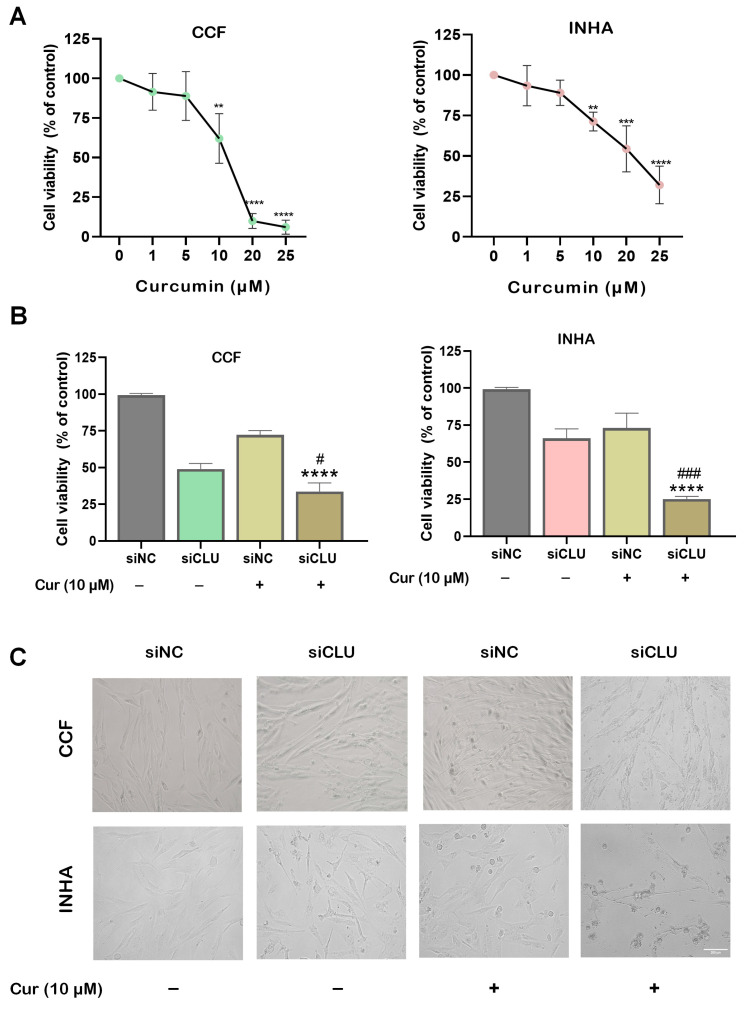
Effect of curcumin on cell viability. Cell viability of control CCF and INHA cells treated with different concentrations (1–25 µM) of curcumin (Cur) for 24 h was determined using the MTT assay (**A**). A comparative analysis of cell viability was then conducted in non-targeting siRNA-transfected (siNC) and CLU siRNA-transfected (siCLU) CCF and INHA cells in the absence or presence of 10 µM curcumin (**B**). Bright-field microscopy micrographs (20× magnification) were taken of untreated cells and those treated with 10 µM curcumin (**C**). Scale bar, 100 µm. Data represent the mean ± SEM of three independent experiments (** *p* < 0.01, *** *p* < 0.001, and **** *p* < 0.0001 vs. corresponding siNC; ^#^ *p* < 0.05 and ^###^ *p* < 0.001 vs. curcumin-untreated).

To investigate the effect of curcumin on the proliferation of CCF and INHA cells, we treated the cells with varying concentrations of curcumin (1 μM, 5 μM, 10 μM, 20 μM, and 25 μM) for 24 h. Cell viability was assessed with an MTT assay. Interestingly, INHA cells exhibited higher viability than CCF cells at the highest concentration of curcumin (Figure 1A), suggesting distinct effects of curcumin on the proliferation of cancerous and non-cancerous astrocytes. To induce senescence, CCF and INHA cells were transfected with siRNA targeting CLU (siCLU) and maintained under these conditions for 72 h. Subsequently, both siCLU-transfected and control (siNC-transfected) cells were treated with 10 μM curcumin for an additional 24 h. A significant reduction in cell viability was observed in siCLU-transfected astrocytes following curcumin treatment (Figure 1B). Additionally, significant morphological changes were observed in siCLU cells treated with curcumin (Figure 1C), indicating early apoptosis. These changes included cell shrinkage, reduced cell size, and increased cytoplasmic density. CLU downregulation was confirmed by immunoblotting (Supplementary Figure S1). The data suggest no direct effect of curcumin on CLU expression. A crystal violet assay was performed to further validate these findings, consistently demonstrating reduced growth in the curcumin-treated siCLU cells (Supplementary Figure S2).

Next, cell migration was evaluated using a scratch wound assay, which revealed that curcumin-treated siCLU cells exhibited a significantly lower migration capacity compared to untreated siCLU cells (Supplementary Figure S3). These results indicate that curcumin may impair cell motility in the context of CLU silencing.

To assess potential recovery in cell growth, we conducted cell counting over a 10-day period. Cell numbers were significantly higher in the siCLU group compared to the curcumin-treated siCLU group (Supplementary Figure S4). These findings indicate that curcumin effectively inhibits cell proliferation in CLU-deficient cells, as reflected by the sustained reduction in cell numbers over time.

### 3.2. Curcumin Mitigates ROS Production in CLU-Deficient Cells

**Figure 2:**
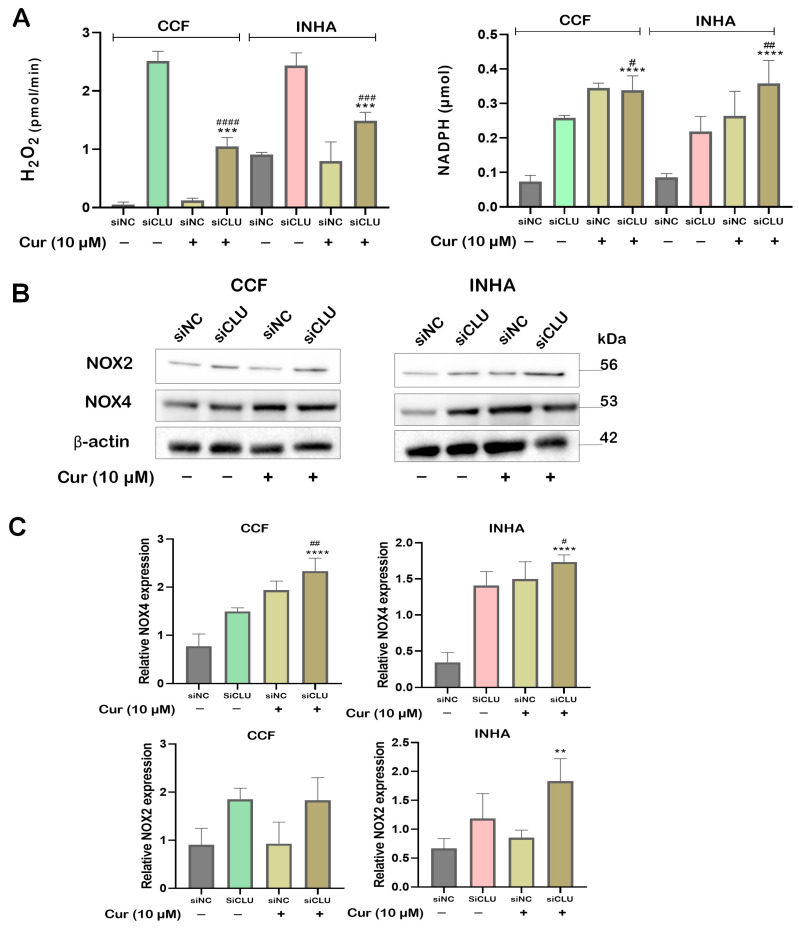
Effect of curcumin on ROS levels and NADPH expression. siNC and siCLU CCF and INHA cells were untreated or treated with 10 µM curcumin (Cur) for 24 h, and hydrogen peroxide (H_2_O_2_) levels were then determined using the Amplex^TM^ Red Hydrogen Peroxide Assay Kit (**A**). NADPH concentration was assessed by a colorimetric assay (**B**), while the expression of NOX4 and NOX2 were analyzed by Western blotting (**C**). Representative Western blot images are shown in the upper panel of (**C**), with their quantitative analysis presented in the lower panel of (**C**). Data represent the mean ± SEM of three independent experiments (** *p* < 0.01, *** *p* < 0.001, and **** *p* < 0.0001 vs. corresponding siNC; ^#^ *p* < 0.05, ^##^ *p* < 0.01, ^###^ *p* < 0.001, and ^####^ *p* < 0.0001 vs. curcumin-untreated).

To investigate the effect of curcumin on reactive oxygen species (ROS) production in CCF and INHA cells, we measured hydrogen peroxide (H_2_O_2_) levels. While curcumin did not significantly affect H_2_O_2_ levels in control cells, it markedly reduced H_2_O_2_ levels in siCLU cells (Figure 2A). This suggests that curcumin may act as an antioxidant by either neutralizing ROS—particularly H_2_O_2_—or enhancing the cell’s detoxification mechanisms. ROS production, including H_2_O_2_, is supported by NADPH, a key cofactor for NADPH oxidase (NOX) enzymes, which facilitate electron transfer to oxygen, leading to the formation of the superoxide anion (O_2_^•−^). Through these pathways, NADPH sustains ROS levels, fueling oxidative reactions and potential cell damage [19]. Interestingly, we found that NADPH was significantly elevated in curcumin-treated siCLU cells (Figure 2B). This suggests that curcumin promotes NADPH accumulation, which in turn supplies electrons to NOX enzymes, specifically NOX2 and NOX4, both of which are crucial for the generation of O_2_^•−^. Activation of NOX2 and NOX4 may further amplify ROS levels, contributing to oxidative stress through cascades that produce secondary reactive species such as H_2_O_2_, thereby intensifying cellular oxidative reactions and potential damage [20]. When examining the expression of NOX2 and NOX4 in curcumin-treated siCLU cells, we found that both were upregulated; however, NOX4 upregulation was more pronounced than that of NOX2 (Figure 2B,C).

The activation of NOX2 and NOX4 by NADPH not only elevates ROS levels but also promotes nitric oxide (NO) production. NO can react with O_2_^•−^ to form peroxynitrite (ONOO^−^), exacerbating oxidative and nitrosative stress, which may contribute to further cellular damage [21]. We did not find any significant changes in NO levels; however, we observed a significant reduction in glutathione levels in curcumin-treated siCLU cells (Supplementary Figure S5). In addition, catalase activity was significantly reduced in the curcumin-treated siCLU group, potentially impairing the cell’s ability to cope with oxidative stress (Supplementary Figure S6A).

### 3.3. Curcumin Induces Apoptosis and Necrosis in CLU-Deficient Cells

**Figure 3:**
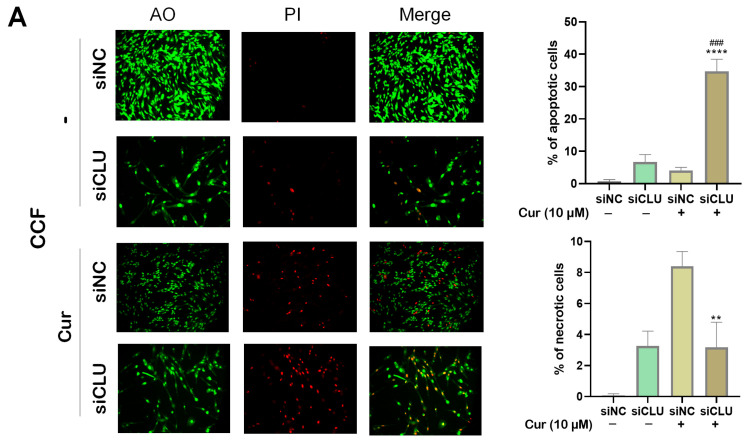

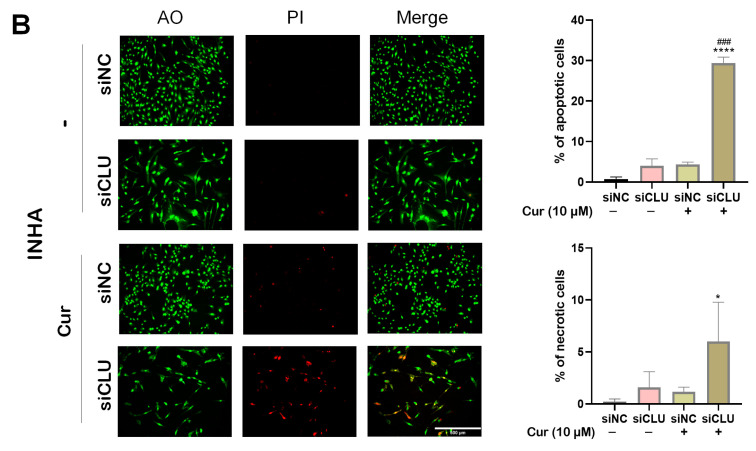
Monitoring cell death processes after curcumin treatment. siNC and siCLU CCF and INHA cells were untreated or treated with 10 µM curcumin (Cur) for 24 h and then stained with propidium iodide (PI) and acridine orange (AO) to assess cell apoptosis and necrosis. Representative immunofluorescence images (20× magnification) of CCF (**A**) and INHA (**B**) cell cultures are shown in the left panels, while the quantitative evaluations of apoptotic and necrotic cell proportions are shown in the right panels. Data represent the mean ± SEM of three independent experiments (* *p* < 0.05, ** *p* < 0.01, and **** *p* < 0.0001 vs. corresponding siNC; ^###^ *p* < 0.001 vs. curcumin-untreated).

**Figure 4:**
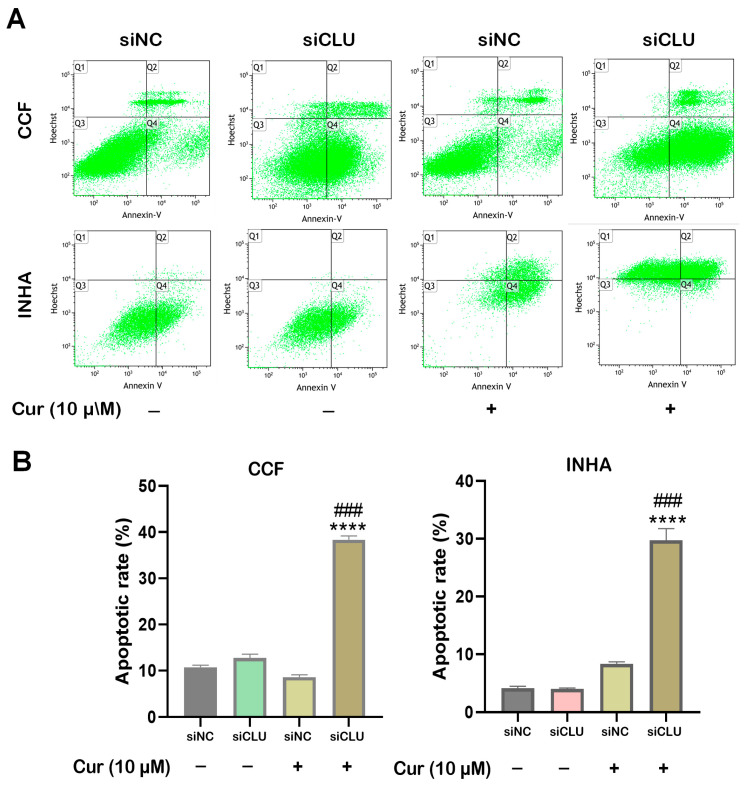
Flow cytometric analysis of apoptosis in curcumin-treated cells. siNC and siCLU CCF and INHA cells were untreated or treated with 10 µM curcumin (Cur) for 24 h. After staining with Annexin V-FITC and Hoechst 33342, the cells were analyzed by flow cytometry. Representative fluorescence dot plots are shown in the upper panel (**A**), while the quantitative evaluations of apoptotic cell distribution are presented in the lower panel (**B**). The four regions in the scattergrams (Q1, Q2, Q3, and Q4) represent different cell populations as determined by flow cytometry. Specifically, Q1 represents dead cells, Q2 late apoptotic cells, Q3 healthy living cells, and Q4 early apoptotic cells. Data represent the mean ± SEM of three independent experiments (**** *p* < 0.0001 vs. corresponding siNC; ^###^ *p* < 0.001 vs. curcumin-untreated).

To investigate the effects of curcumin on cell death in CCF and INHA cells, we performed fluorescence microscopy using acridine orange (AO) and propidium iodide (PI) staining. AO stains live cells with intact membranes, resulting in green fluorescence, while PI stains dead or late apoptotic cells with compromised membranes, producing red fluorescence. Our results demonstrated that curcumin treatment significantly increased the number of red-fluorescent cells in siCLU cells, indicating the induction of necrosis and late-stage apoptosis. In contrast, the control group exhibited minimal PI staining, suggesting a low level of necrosis or late apoptosis. Additionally, AO staining revealed a reduction in the number of viable (green-fluorescent) cells following curcumin treatment, further supporting the induction of cell death (Figure 3A,B). These findings were confirmed by flow cytometric analysis of apoptosis, which showed a similar trend (Figure 4A,B). Collectively, these data suggest that curcumin effectively induces both apoptosis and necrosis in CLU-deficient cells, highlighting its potential to promote cell death through multiple mechanisms.

### 3.4. Curcumin Induces Caspase Activation in CLU-Deficient Cells

**Figure 5:**
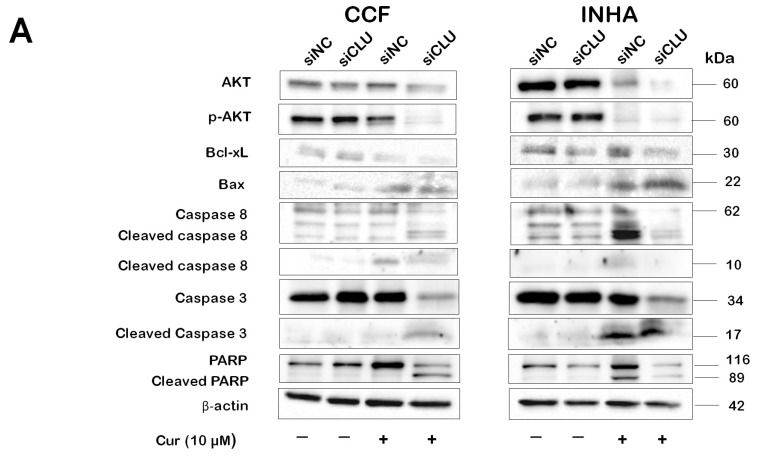

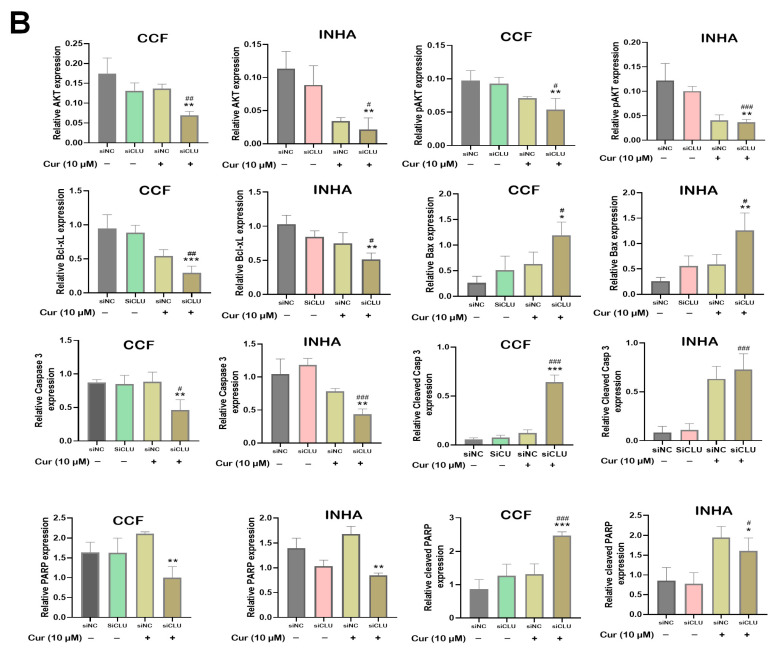
Effects of curcumin on AKT and apoptotic protein expression. siNC and siCLU CCF and INHA cells were untreated or treated with 10 µM curcumin (Cur) for 24 h. Protein expression levels of AKT, p-AKT, and selected apoptotic proteins (Bcl, Bcl-xL, caspase 8, caspase 3, and PARP) were analyzed by Western blotting. Representative Western blot images are shown in the upper panel (**A**), while their quantitative evaluations are presented in the lower panel (**B**). Data represent the mean ± SEM of three independent experiments (* *p* < 0.05 and ** *p* < 0.01, and *** *p* < 0.001 vs. corresponding siNC; ^#^ *p* < 0.05, ^##^ *p* < 0.01, and ^###^ *p* < 0.001 vs. curcumin-untreated).

The expression of AKT and selected apoptotic proteins in CCF and INHA cells under different experimental conditions was assessed by Western blot analysis (Figure 5). We found that curcumin treatment led to a decrease in both phosphorylated AKT (pAKT) and total AKT levels. Similarly, the expression of STAT3 and its phosphorylated form was also markedly reduced by curcumin (Supplementary Figure S6B). The downregulation of AKT and STAT3 signaling following curcumin treatment suggests an inhibition of pro-survival signaling, thereby facilitating the activation of apoptotic pathways. Next, we examined the expression of cleaved caspase-8, caspase-9, and caspase-3. Cleaved caspase-8, caspase-9, and caspase-3 were detected in curcumin-treated cells. Interestingly, cleaved caspase-3 was present only in curcumin-treated INHA cells and curcumin-treated CLU-deficient CCF cells. In INHA cells, cleaved caspase-3 was observed in both the curcumin-treated siNC and siCLU groups. Similarly, cleaved caspase-8 was expressed in both the curcumin-treated siNC and siCLU groups of CCF and INHA cells. Additionally, we investigated the expression of PARP, a downstream effector in the caspase pathway. In CCF cells, cleaved PARP was detected only in curcumin-treated siCLU cells, with no expression in the siNC group. In contrast, cleaved PARP was observed in both the curcumin-treated siNC and siCLU groups of INHA cells. Furthermore, we observed a decrease in Bcl-xL expression and an upregulation of Bax protein in the curcumin-treated groups of both CCF and INHA cells.

### 3.5. Curcumin Induces Autophagy in CLU-Deficient Cells

**Figure 6:**
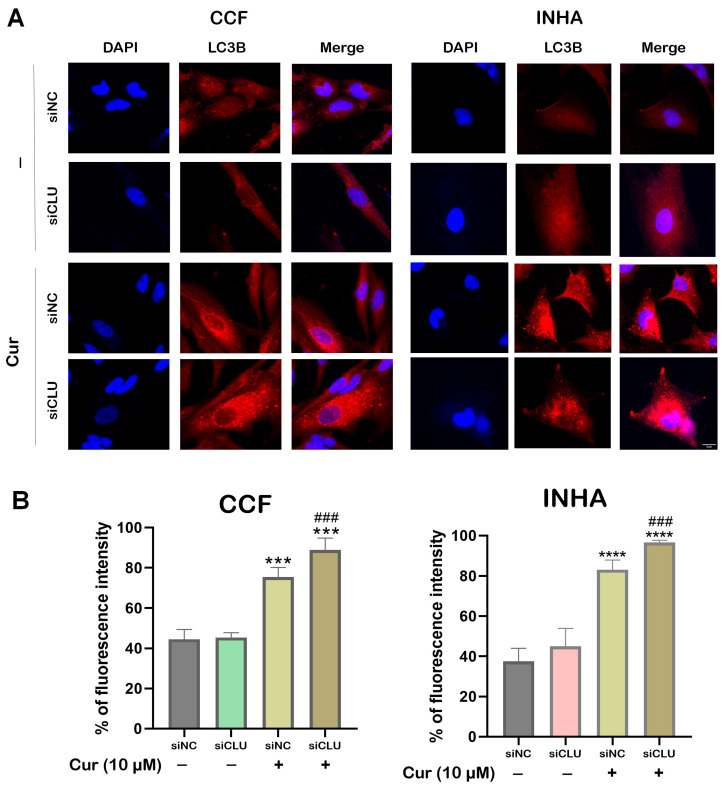
Effect of curcumin on autophagy. siNC and siCLU CCF and INHA cells were untreated or treated with 10 µM curcumin (Cur) for 24 h and autophagy was then assessed using ImageJ. Representative immunofluorescence images (20× magnification) of cell cultures are shown in the upper panels (**A**), while the quantitative evaluations of the proportion of autophagic cells are presented in the lower panels (**B**). Data represent the mean ± SEM of three independent experiments (*** *p* < 0.001 and **** *p* < 0.0001 vs. corresponding siNC; ^###^ *p* < 0.001 vs. curcumin-untreated).

To assess the impact of curcumin on autophagy in CLU-deficient senescent cells, we performed immunofluorescence staining for LC3B, a key marker of autophagosome formation. LC3B is widely used to monitor autophagy as it localizes to autophagic membranes and accumulates during autophagosome formation [11]. Immunofluorescence analysis revealed a significant increase in LC3B puncta in curcumin-treated siCLU cells compared to untreated siCLU groups (Figure 6A,B). This increase suggests enhanced autophagosome formation, indicating that curcumin stimulates autophagy in these cells. Quantification of LC3B-positive puncta per cell showed a marked rise in autophagic activity upon curcumin treatment, consistent with an increased autophagic flux.

## 4. Discussion

Glioma remains one of the most aggressive and treatment-resistant forms of brain cancer, presenting significant therapeutic challenges despite advances in surgery, radiotherapy, and chemotherapy. The heterogeneous nature of gliomas and their high invasiveness and resistance to apoptosis complicate effective treatment. CLU, a glycoprotein involved in cell survival and apoptosis resistance, is commonly overexpressed in several cancers and gliomas [22,23]. It acts as a chaperone protein, assisting in cellular responses to stress and often promoting pro-survival pathways that make cancer cells more resistant to treatment. High levels of CLU are associated with therapy resistance and poor prognosis in several cancers [12]. Silencing CLU expression through RNA interference or other gene silencing technologies has shown promise in sensitizing cancer cells to treatment by decreasing cell viability and enhancing apoptosis, thus disrupting the cellular environment conducive to tumor survival [11,24]. The silencing of CLU has been demonstrated to induce senescence in pancreatic cancer cells. Consistent with this, our previous study also observed similar effects in promoting senescence in gliomas following CLU silencing [13,14]. Curcumin, a bioactive compound derived from Curcuma longa, has recently garnered attention as a potential adjuvant in cancer therapy due to its multi-targeted anticancer properties [24,25,26]. Curcumin exhibits strong anticancer properties by modulating cell signaling pathways involved in apoptosis, cell cycle regulation, and metastasis [27]. It inhibits key cancer-related pathways, such as NF-κB, PI3K/AKT, and STAT3, thereby promoting apoptosis and reducing cancer cell proliferation. The PI3K/AKT pathway is frequently hyperactivated in glioma, promoting survival and growth, and inhibiting apoptosis [28]. Curcumin’s inhibitory effect on AKT phosphorylation can thus disrupt these survival signals, particularly when CLU, a survival-promoting protein, is also downregulated. This dual mechanism could synergistically enhance cancer cell apoptosis and reduce tumor growth.

In the present study, we first found that curcumin exerts differential effects on the proliferation of cancerous and non-cancerous astrocytes. While INHA cells exhibited higher viability than CCF cells at elevated curcumin concentrations, CLU silencing significantly sensitized cells to curcumin-induced growth inhibition. The reduction in cell viability and the morphological changes observed in siCLU-transfected cells following curcumin treatment highlight its potential role in suppressing tumor cell proliferation. Furthermore, the impaired migration of curcumin-treated siCLU cells suggests that CLU may contribute to cell motility, and its downregulation enhances curcumin’s inhibitory effects. The sustained reduction in cell numbers over a 10-day period reinforces curcumin’s long-term impact on CLU-deficient cells. These results provide insights into curcumin’s therapeutic potential, particularly in targeting CLU-associated pathways in glioma.

It is known that curcumin inhibits malignancy by inducing ROS [29]. Our results showed a significant increase in H_2_O_2_ production in curcumin-treated siCLU cells compared to the curcumin-treated siNC group, suggesting an enhanced oxidative stress response. This was accompanied by the upregulation of NOX [30], indicating a potential mechanism contributing to elevated ROS levels. Additionally, the combined treatment led to a marked depletion of glutathione, a key antioxidant involved in ROS neutralization. The reduction in GSH levels suggests a heightened demand for antioxidant defense [31], ultimately weakening the cellular antioxidant system and increasing susceptibility to oxidative stress. Consistent with previous studies [32], we found that catalase activity was notably reduced in the curcumin-treated siCLU group, further impairing the cell’s ability to detoxify H_2_O_2_ and exacerbating oxidative stress. This oxidative imbalance may further amplify cellular damage, potentially contributing to curcumin’s inhibitory effects on cell proliferation in CLU-deficient cells. The accumulation of ROS can trigger various stress pathways, leading to cellular damage, apoptosis, and autophagy, ultimately impairing cancer cell survival [33]. Our findings highlight the role of oxidative stress in curcumin-mediated cytotoxicity and suggest that targeting redox homeostasis may enhance its therapeutic potential in glioma treatment. The dual effect of CLU silencing and curcumin-induced ROS suggests a synergistic mechanism where oxidative stress acts as a mediator of cytotoxicity, thus contributing to the inhibition of cancer cell growth. These findings underscore the importance of oxidative stress in the therapeutic action of curcumin and support its potential as an adjuvant in cancer treatments targeting CLU pathways.

Our Western blot analysis revealed a decrease in pAKT expression in curcumin-treated cell groups, consistent with previous studies demonstrating curcumin’s effect on glioma proliferation via inhibition of the pAKT pathway [34]. The reductions in both total AKT and pAKT levels suggest that curcumin inhibits pro-survival signaling, thereby promoting apoptotic pathways. Since active AKT phosphorylates and inhibits pro-apoptotic proteins like Bad while activating anti-apoptotic proteins such as Bcl-2 [35], its downregulation may disrupt these survival mechanisms and enhance apoptosis. Consistently, our data showed a reduction in Bcl-xL expression in curcumin-treated cells, accompanied by an increase in the pro-apoptotic protein Bax. Furthermore, by lowering pAKT levels, curcumin disrupts these protective mechanisms, leading to the activation of caspases—key enzymes involved in the execution of apoptosis [36]. This shift toward caspase activation and subsequent cell death is consistent with our data, as we observed a reduction in cell viability and enhanced apoptosis in curcumin-treated cells. Caspases are key proteolytic enzymes that initiate apoptosis by triggering a cascade of events, leading to controlled cell dismantling through targeted protein cleavage [37]. In our study, Western blot analysis was employed to assess the expression of cleaved caspase-8, caspase-9, and caspase-3. Cleaved caspase-8, caspase-9, and caspase-3 were detected in curcumin-treated cells. In CCF cells, however, cleaved caspase-3 was only present in siCLU-treated cells exposed to 10 µM of curcumin, with no expression observed in control siNC-treated cells. In INHA cells, cleaved caspase-3 was observed in both curcumin-treated siNC and siCLU groups. This suggests that in CCF cells, the presence of cleaved caspase-3 in siCLU-treated cells exposed to curcumin may be specifically associated with CLU downregulation. In contrast, in INHA cells, curcumin treatment alone appears sufficient to trigger caspase-3 activation, regardless of CLU silencing. These findings indicate that cancer cells exhibit resistance to normal apoptosis induction, and a combined treatment approach could potentially enhance therapeutic efficacy. Cleaved caspase-8 was expressed in both curcumin-treated siNC and siCLU groups in CCF and INHA cells. Additionally, we investigated the expression of PARP, a downstream target in the caspase pathway that is cleaved upon caspase activation [38]. In the CCF cell group, cleaved PARP was detected only in curcumin-treated siCLU cells, with no expression in the siNC group. This suggests that in CCF cells, caspase pathway activation, as indicated by cleaved PARP expression, is specifically linked to CLU downregulation in response to curcumin treatment. The absence of cleaved PARP in the siNC-treated group further supports the idea that CLU plays a critical role in regulating caspase-mediated apoptosis in this context. In contrast, cleaved PARP was observed in both the curcumin-treated siNC and siCLU groups in INHA cells. These results suggest that combined CLU silencing and treatment with curcumin could significantly reduce cell proliferation in cancer cells.

In this study, we also found that curcumin treatment in CLU-silenced cells not only enhanced apoptosis but also induced autophagy, as evidenced by the upregulation of LC3B, a key marker of autophagic activity [39]. This suggests that autophagy acts as an additional mechanism alongside apoptosis in the response to curcumin, as evidenced from previous studies [24] and CLU silencing. While curcumin-induced apoptosis plays a central role in cell death, the induction of autophagy may serve as a protective mechanism or a supplementary process to manage cellular stress [40]. These findings underscore the interplay between apoptosis and autophagy in curcumin-treated CLU-silenced cells, offering new insights into potential therapeutic strategies for GBM and other cancers. In our study, the combination of curcumin and CLU silencing presents a promising therapeutic strategy. By silencing CLU, glioma cells lose a major defense against apoptosis, thereby becoming more vulnerable to treatments that promote cell death. Curcumin’s role in activating caspases and downregulating anti-apoptotic proteins, such as Bcl-2, aligns well with this approach, further tilting the balance towards apoptosis [41]. Previous studies have shown that curcumin can enhance the effects of other therapeutic agents when tumor survival pathways are simultaneously targeted [42]. We hypothesize that the combination treatment in glioma models may promote enhanced cell death by triggering caspase activation, downregulating the AKT signaling pathway, inducing autophagy, and reducing tumor proliferation resistance.

The elevated autophagy observed in the curcumin-treated group suggests that curcumin may activate the autophagic process as a mechanism to counteract stress or cellular damage in the context of CLU silencing. Overall, these findings demonstrate that curcumin induces autophagy in siCLU cells, as evidenced by the increased presence of LC3B-labeled autophagosomes, highlighting its potential role in modulating autophagic pathways under conditions of altered CLU expression.

Although the current study is based on in vitro experiments, the comparison between the treatment and control groups provides valuable insights into the potential clinical relevance of the findings. The treatment group exhibited significant effects, including reduced cell viability, inhibition of tumor cell proliferation, and induction of apoptosis—suggesting promising therapeutic potential. In contrast, the control group showed expected behavior, such as continued proliferation and minimal changes in survival pathways. While these results are encouraging, they serve primarily as a foundation for future in vivo studies and clinical trials necessary to evaluate the true clinical efficacy of the treatment. It is important to emphasize that although in vitro studies are critical for understanding underlying mechanisms, their translational value can only be confirmed through rigorous testing in animal models and ultimately in human patients.

Curcumin has shown promising anticancer effects in vitro, but several challenges limit its clinical application. Chief among these are its poor bioavailability, low aqueous solubility, rapid metabolic degradation, and swift systemic elimination, all of which significantly diminish its therapeutic efficacy in vivo [43]. Curcumin’s limited ability to cross biological barriers—particularly the blood–brain barrier—further complicates its use in treating CNS malignancies such as glioma [44,45]. To overcome the lipid barrier, several formulation strategies have been explored, including liposomes, micelles, nanoemulsions, solid lipid nanoparticles, and phospholipid complexes [46]. These advanced delivery systems enhance curcumin’s solubility, protect it from rapid degradation, and facilitate its transport across lipid membranes, thereby improving cellular absorption and therapeutic efficacy. Although this study did not utilize such delivery systems, the promising in vitro results provide a strong rationale for incorporating these strategies in future research to enhance curcumin’s bioavailability and clinical applicability.

CLU silencing holds significant promise in the context of tumor biology due to its multifaceted role in cancer progression, therapy resistance, and metastasis. CLU is often overexpressed in various cancers, contributing to cell survival, evasion of apoptosis, and adaptation to stress conditions, such as hypoxia and oxidative damage [47]. This overexpression helps tumor cells resist the cytotoxic effects of chemotherapy and radiation, leading to treatment resistance and poorer patient outcomes. By silencing CLU, the protective mechanisms that enable tumor cells to survive and proliferate under hostile conditions can be disrupted [22]. In addition to its role in survival pathways, CLU is involved in promoting epithelial–mesenchymal transition, a critical process in metastasis [48]. Silencing CLU may reduce the ability of tumor cells to detach, invade, and migrate to distant tissues, thus limiting the spread of cancer. The novelty of CLU silencing in cancer therapy lies in its ability to target the cellular mechanisms underlying both the treatment resistance and metastatic potential of tumors, offering a strategy that goes beyond traditional chemotherapy. Unlike conventional treatments that focus on killing rapidly dividing cells, CLU silencing could potentially restore tumor cells to a more vulnerable state, making them more susceptible to other therapies and reducing the likelihood of relapse [15,49]. This approach has the potential to overcome current therapeutic limitations, providing a more targeted and effective solution for tumors resistant to conventional treatments.

## 5. Conclusions

The combination of curcumin and CLU silencing may offer a novel therapeutic approach in gliomas, harnessing the apoptosis-promoting and survival-inhibiting effects of both treatments. While preclinical evidence supports this strategy, further investigation is needed to optimize curcumin delivery and assess the long-term safety and efficacy of CLU silencing in cancer therapy. If successful, this combination could pave the way for more effective, targeted therapies, such as receptor-specific inhibitors or nanoparticle-based drug delivery systems for the treatment of gliomas and other therapy-resistant cancers. Our findings demonstrate that CLU silencing significantly enhances the effect of curcumin, as evidenced by a marked reduction in cell viability. This effect is likely mediated through the induction of apoptosis and autophagy, as well as the downregulation of key survival pathways, including the AKT signaling pathway. These results suggest that the synergistic combination of CLU silencing and curcumin treatment could amplify the anticancer effects in glioma cells, offering a promising therapeutic strategy.

## 6. Future Directions

Given the encouraging in vitro results with the combined use of curcumin and CLU inhibition, future studies should prioritize translating these findings into in vivo models to assess their therapeutic efficacy in a more physiologically relevant context. Animal models of tumorigenesis will be essential for evaluating not only the anti-tumor activity but also the pharmacokinetics, systemic toxicity, and the long-term effects of this combination approach. One major limitation of curcumin remains its poor solubility and limited bioavailability, which can hinder its clinical application. Therefore, developing advanced drug delivery systems—such as nanoparticle-based formulations, liposomes, or curcumin analogs—should be a key focus to enhance its absorption, stability, and tumor-targeted delivery. Moreover, future research should aim to elucidate the molecular interactions between curcumin and CLU signaling pathways. CLU is involved in several tumor-promoting processes, including apoptosis resistance, stress adaptation, and metastasis. Curcumin, known to modulate multiple signaling cascades such as NF-κB, PI3K/AKT, and MAPK, may synergize with CLU silencing to amplify pro-apoptotic or anti-proliferative effects. Understanding these interactions at a mechanistic level will be crucial for identifying biomarkers of response and potential resistance pathways. Additionally, exploring this combination in different cancer subtypes or therapy-resistant models could broaden its applicability. Ultimately, this line of investigation may contribute to the development of a novel, multi-targeted therapeutic strategy with enhanced clinical efficacy and improved patient outcomes.

## Data Availability

The original contributions presented in this study are included in the article. Further inquiries can be directed to the corresponding author.

## Supplementary Materials

The following supporting information can be downloaded at https://www.mdpi.com/article/10.3390/pharmaceutics17060679/s1: Figure S1: Western blot analysis of CLU expression levels; Figure S2: Visualization of the impact of curcumin on cell survival and growth arrest; Figure S3: Scratch wound healing assay; Figure S4: Effect of curcumin on cell numbers during prolonged cultivation of CCF and INHA cells; Figure S5: Effect of curcumin on GSH levels; Figure S6: Effect of curcumin on STAT and catalase expression; Supplementary Material-WB: Examples of original images of immunoblots and Ponceau staining.
