# Supplementary material for: Curcumin-Induced Apoptotic Cell Death in Human Glioma Cells Is Enhanced by Clusterin Deficiency
Source: Pharmaceutics. 2025 May 22;17(6):679. doi: 10.3390/pharmaceutics17060679 (PMC12195681; doi:10.3390/pharmaceutics17060679)
Supplement: Supplementary file 1 [file pharmaceutics-17-00679-s001.zip › Supplementary Figs.pdf]

Supplementary Figures

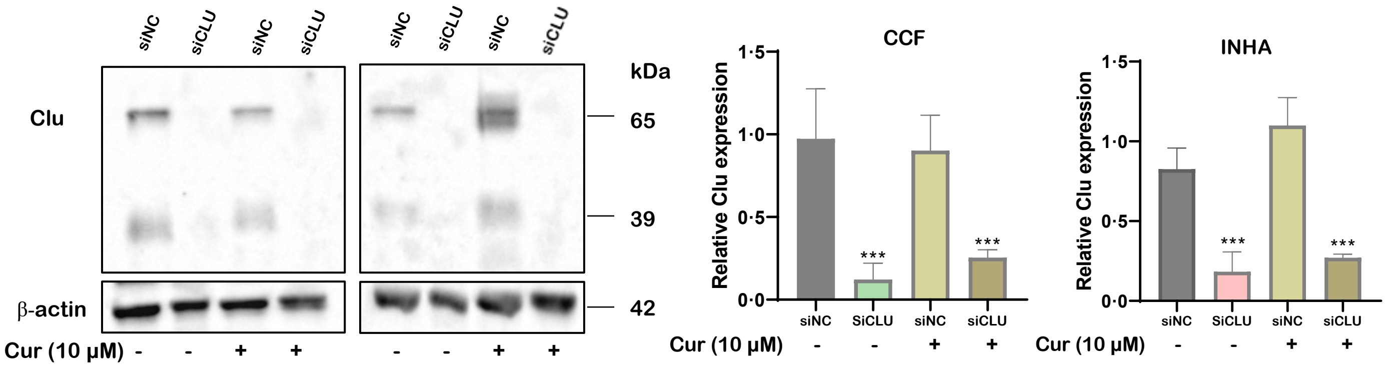

Supplementary Figure S1. Western blot analysis of CLU expression levels. Western blot analysis was performed to assess CLU expression levels in CCF and INHA cells transfected with siNC or siCLU, followed by treatment with 10 μM curcumin (Cur) for 24 hours. Representative Western blot images are shown in panel (A), while their quantitative evaluation is presented in panel (B), demonstrating effective CLU knockdown and no significant direct effect of curcumin treatment on CLU expression. Data represent the mean ± SEM of three independent experiments (****p* < 0.001 *vs* corresponding siNC).

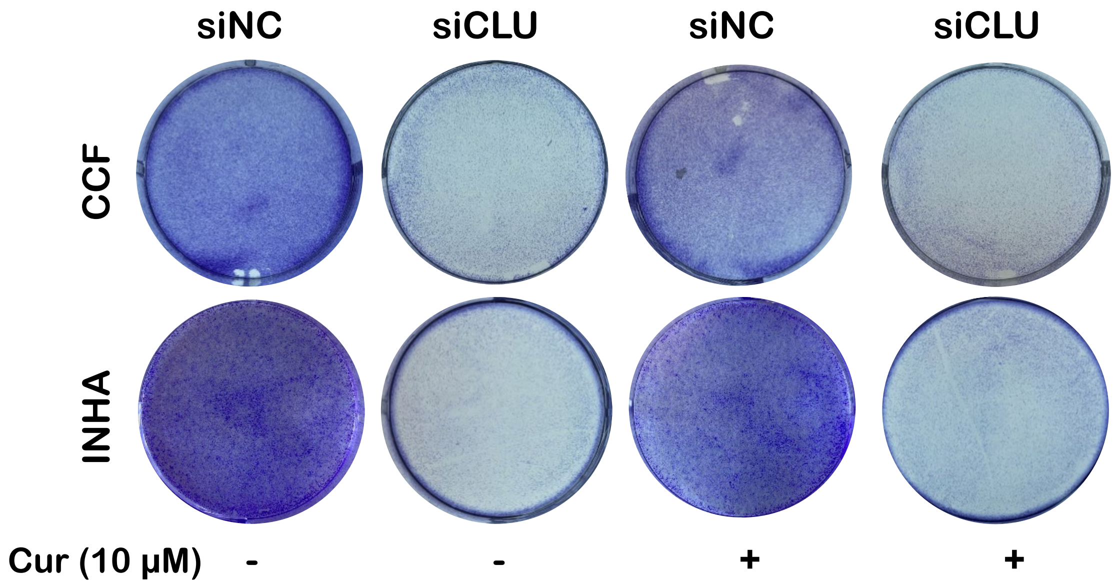

Supplementary Figure S2. Visualization of the impact of curcumin on cell survival and growth arrest. siNC and siCLU CCF and INHA cells were untreated or treated with 10 μM curcumin (Cur) for 24 hours, followed by staining with crystal violet to evaluate cell proliferation and viability. The amount of dye bound to the cells correlates with the cell number, providing visual representation cell survival.

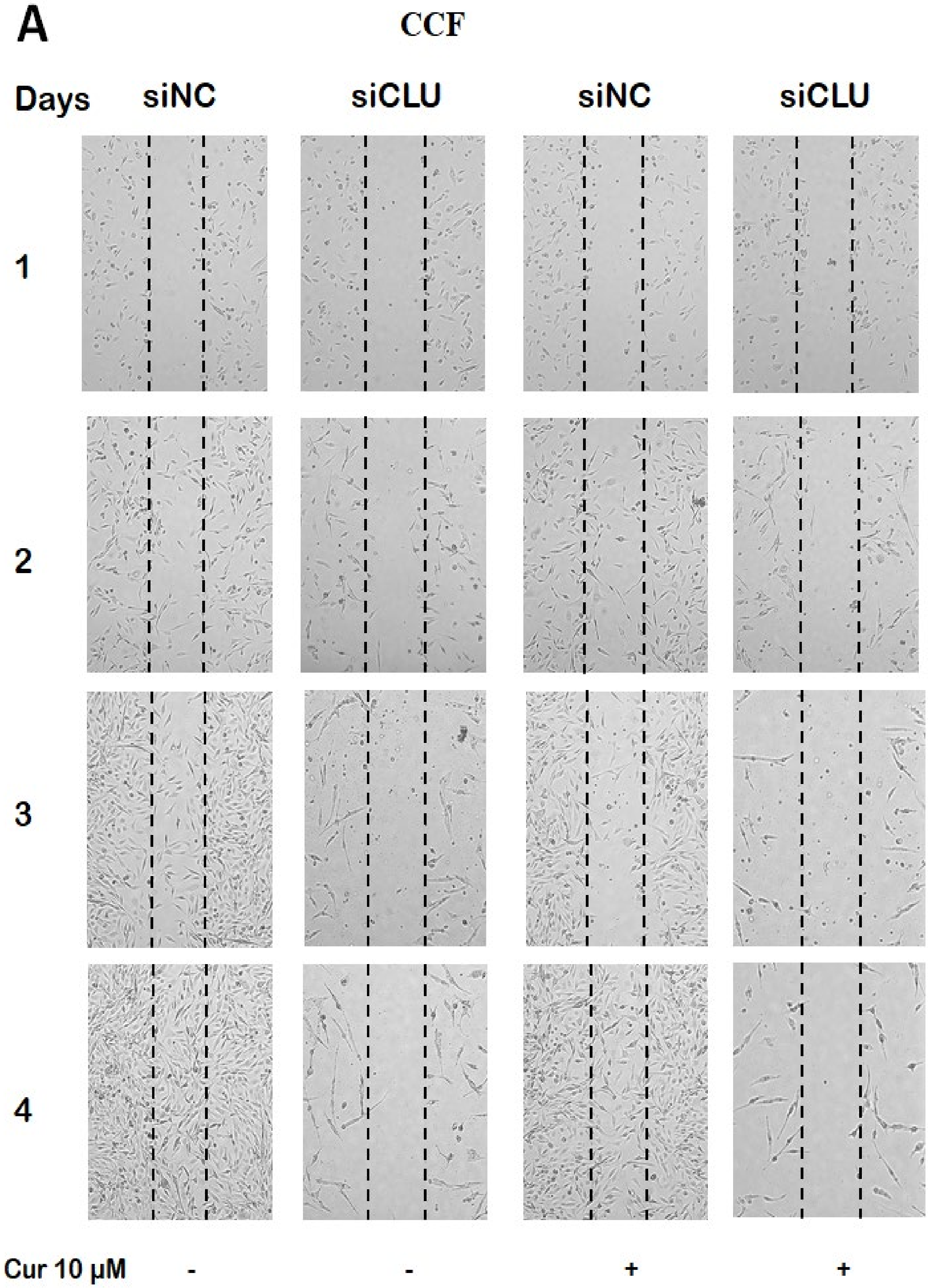
A
CCF
Days
siNC
siCLU
siNC
siCLU
1
2
3
4
Cur 10 μM
-
-
+
+

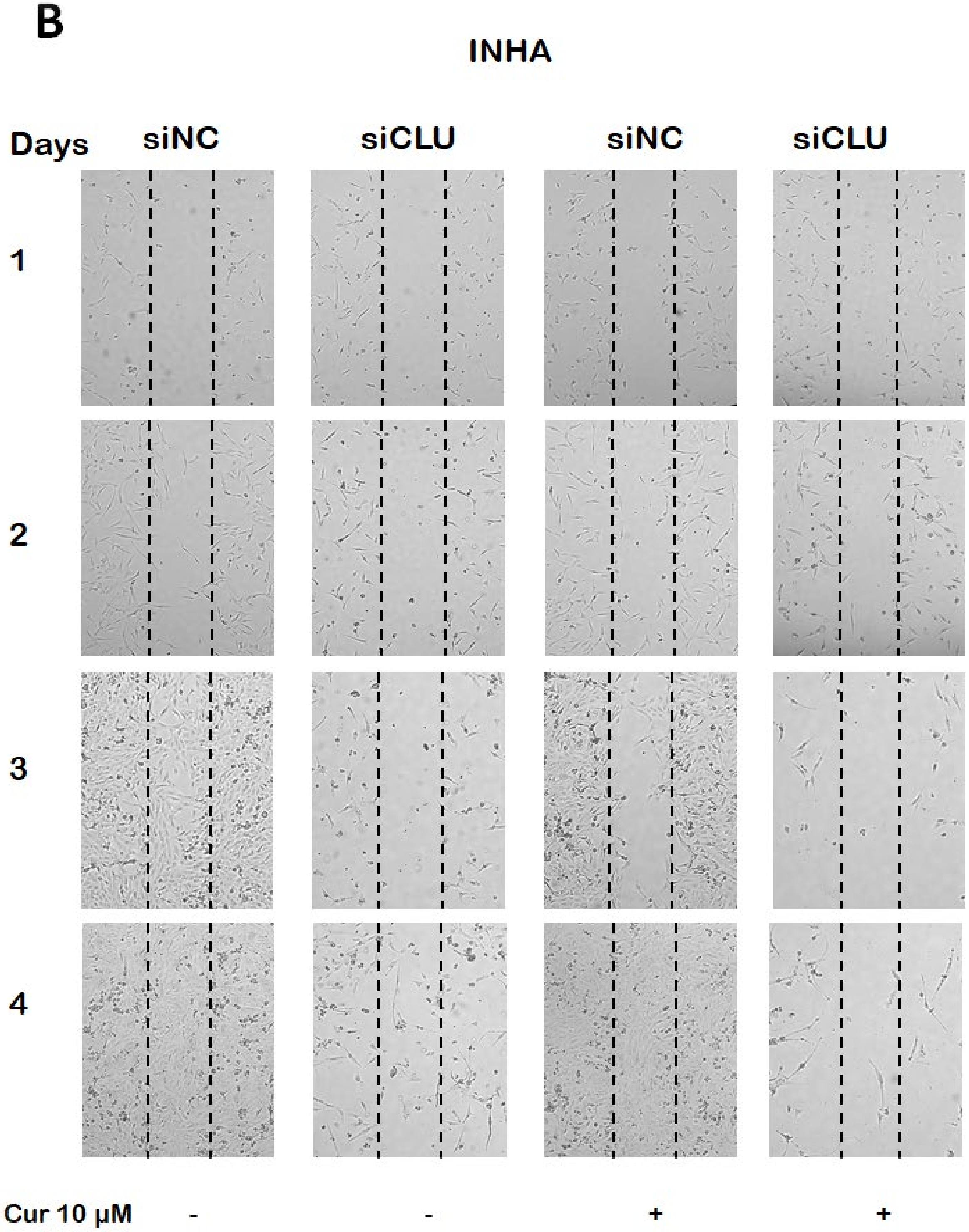
B
INHA
Days
siNC
siCLU
siNC
siCLU
1
2
3
4
Cur 10 μM
-
-
+
+

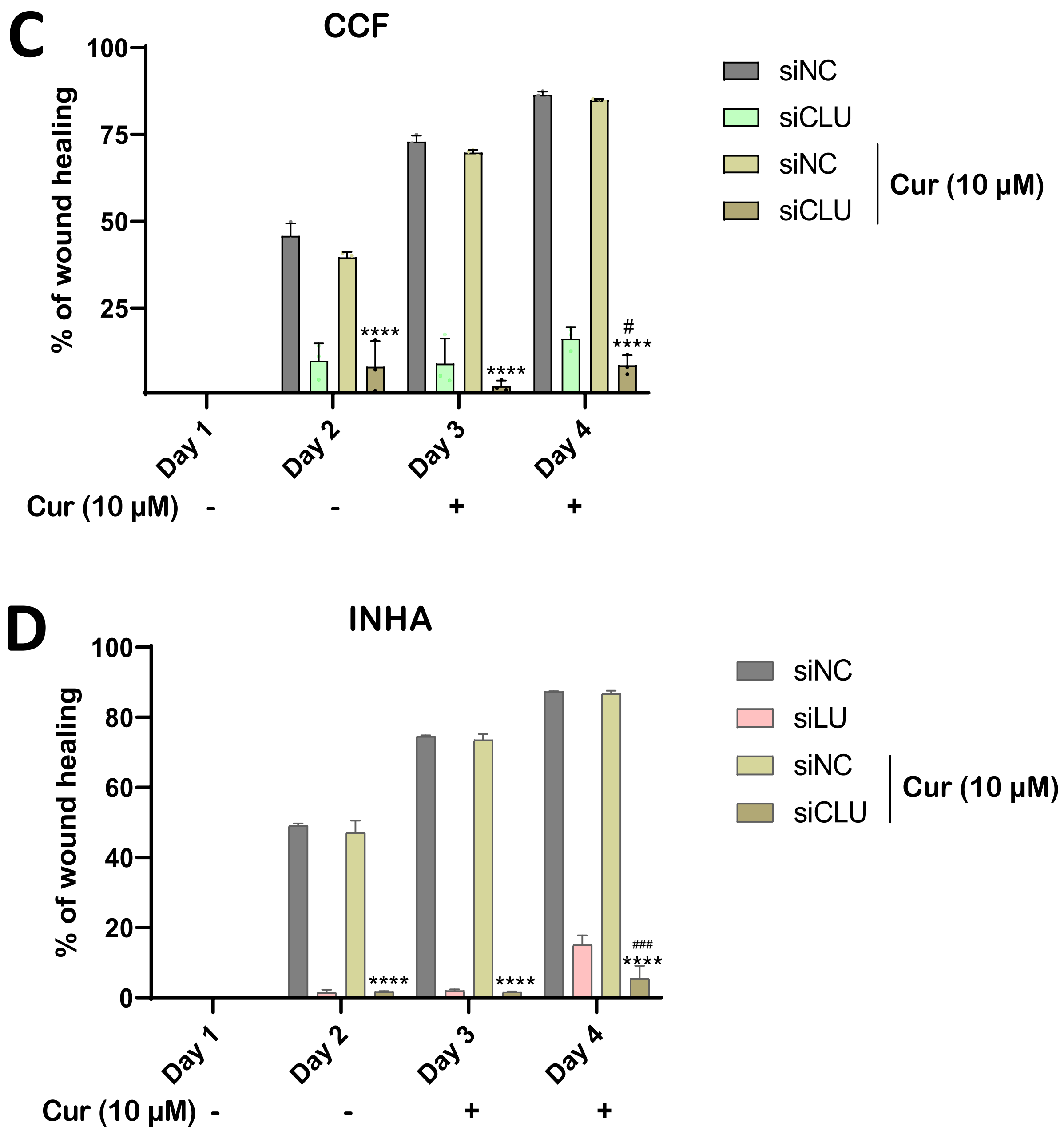

Supplementary Figure S3. Scratch wound healing assay. Cells were reverse transfected with siNC and siCLU. After 4 hours of incubation, a straight-line scratch was created using a 1-mm pipette tip. Images of the cell cultures were captured every 24 hours using a phase-contrast microscope at 4× magnification. On the third day, cells were exposed to curcumin for 24 hours, and images were taken on the fourth day (A and B). The width of the scratch wound was measured using ImageJ. Wound healing, which reflects the rate of cell migration, was assessed from three independent experiments and expressed as a percentage (C and D). Data represent the mean ± SEM of three independent experiments (****p < 0.0001 *vs* corresponding siNC; $^{\#}p < 0.05$, $^{\#\#\#}p < 0.001$ *vs* curcumin-untreated).

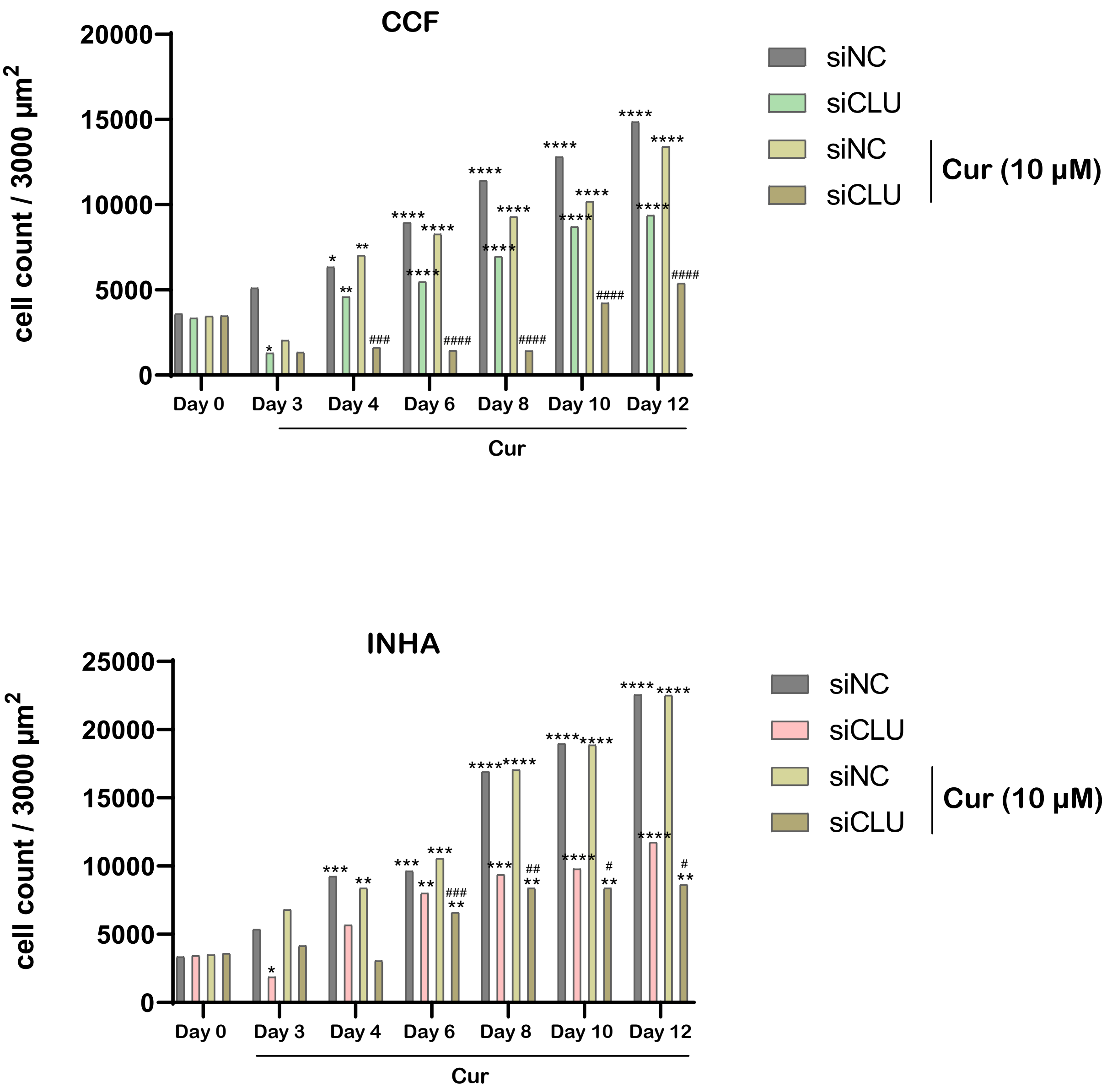

Supplementary Figure S4. Effect of curcumin on cell number during prolonged cultivation of CCF and INHA cells. siNC and siCLU CCF and INHA cells were untreated or treated with 10 μM curcumin (Cur) and maintained for additional 12 days to monitor cell numbers using automated cell counting (using Agilent BioTek Cytation 5). Growth arrest in siCLU-transfected cells lasted until day 4, after which proliferation resumed. However, in curcumin-treated siCLU groups, proliferation did not resume after day 4. Data represent the mean ± SEM of three independent experiments (*p < 0.05, **p < 0.01, ***p < 0.001 and ****p < 0.0001 *vs* day 0; $^{\#}p < 0.05$, $^{\#\#}p < 0.01$, $^{\#\#\#}p < 0.001$ and $^{\#\#\#\#}p < 0.0001$ *vs* curcumin-untreated).

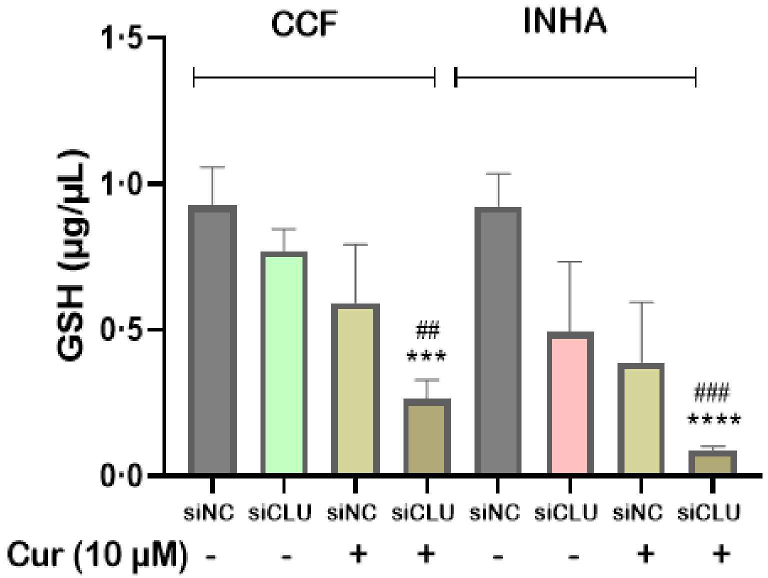

Supplementary Figure S5. Effect of curcumin on GSH level. siNC and siCLU CCF and INHA cells were untreated or treated with 10 µM curcumin (Cur) for 24 hours. Cell samples were then analysed for nitrite concentration using the Reduced Glutathione Assay Kit (Abcam). Data represent the mean ± SEM of three independent experiments (***p < 0.001, ****p < 0.0001 *vs* corresponding siNC; $^{\#\#}p$ < 0.01, $^{\#\#\#}p$ < 0.001 *vs* curcumin-untreated).

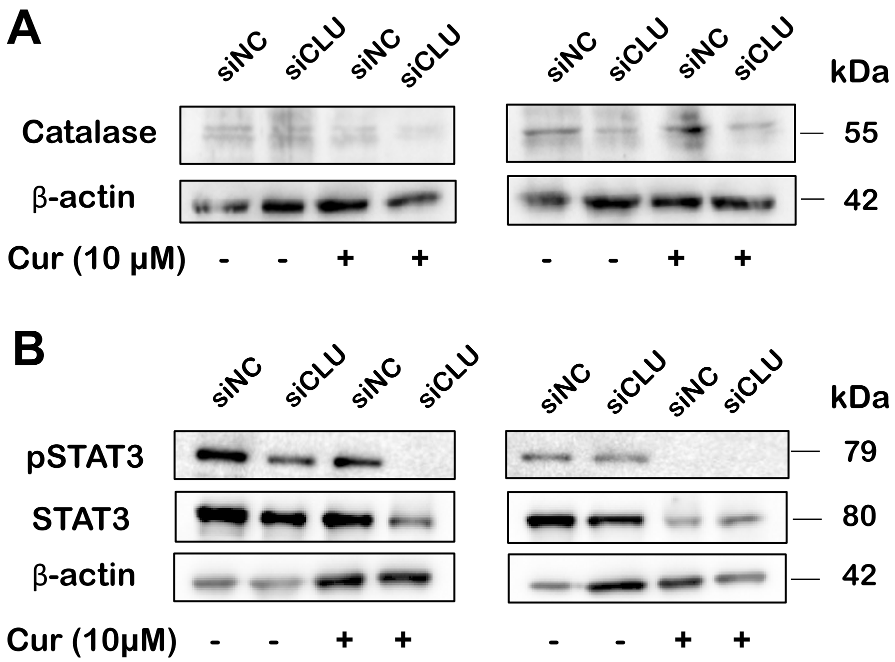

Supplementary Figure S6. Effect of curcumin on catalase (A) and STAT (B) expression. siNC and siCLU CCF and INHA cells were untreated or treated with 10 µM curcumin (Cur) for 24 hours. Expression levels of catalase, STAT, and p-STAT, were then assessed by Western blot analysis.
