# Supplementary material for: Curcumin-Induced Apoptotic Cell Death in Human Glioma Cells Is Enhanced by Clusterin Deficiency
Source: Pharmaceutics. 2025 May 22;17(6):679. doi: 10.3390/pharmaceutics17060679 (PMC12195681; doi:10.3390/pharmaceutics17060679)
Supplement: Supplementary file 1 [file pharmaceutics-17-00679-s001.zip › Supplementary Material - WB.pdf]

Supplementary Material

Examples of original images of immunoblots and Ponceau staining. CCF and INHA cells were treated with siRNA against CLU (siCLU) or scrambled siRNA (siNC) for 72 hours, and then either with or without curcumin (10 μM) for 24 hours. Cell homogenates were immunoblotted with antibodies against NOX4 (56 kDa), NOX2 (53 kDa), AKT and pAKT (60 kDa), Bcl-xl (30 kDa), Bax (22 kDa), caspase 8 (62 kDa) and cleaved caspase 8 (10 kDa), caspase 3 (34 kDa) and cleaved caspase 3 (17 kDa), PARP (116 kDa) and cleaved PARP (89 kDa). Before blocking and probing with antibodies, the nitrocellulose membranes were stained with Ponceau S to normalize protein loading. Shown are examples of Western blots (A) and Ponceau S-stained membranes (B).

NOX4

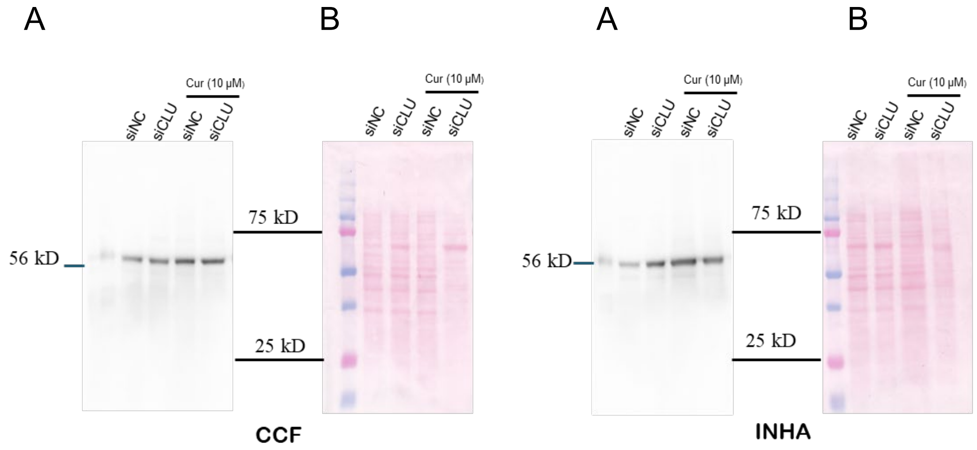

NOX2

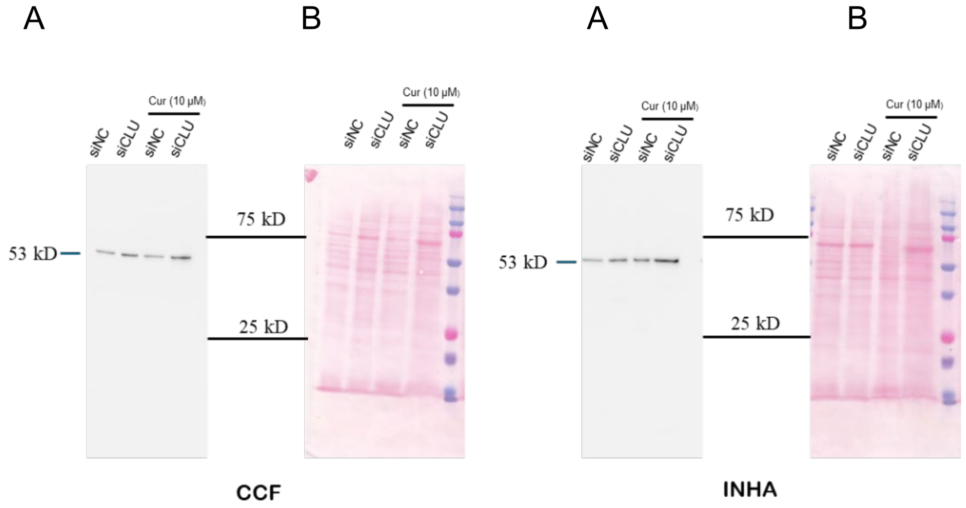

AKT

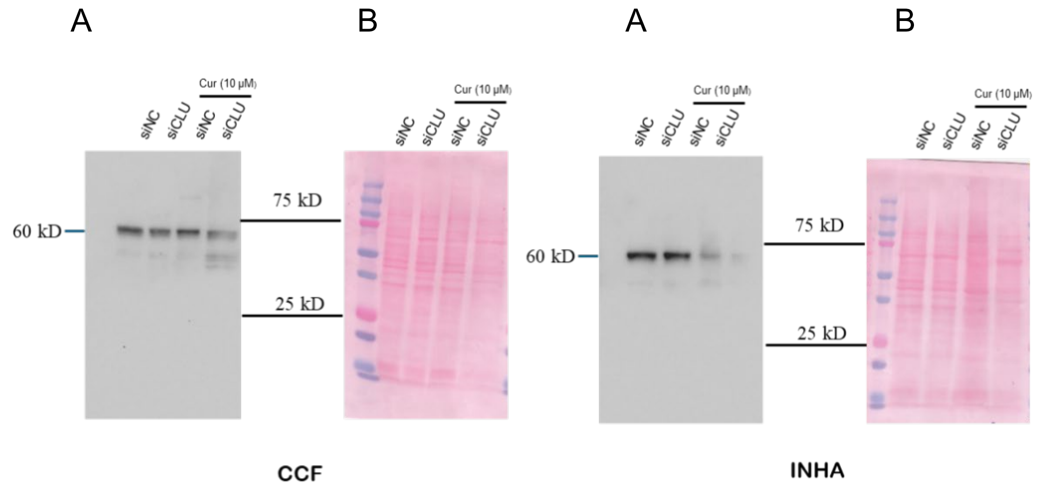
A
B
A
B
Cur (10 μM)
siNC
siCLU
siNC
siCLU
60 kD
75 kD
25 kD
CCF
INHA

pAKT

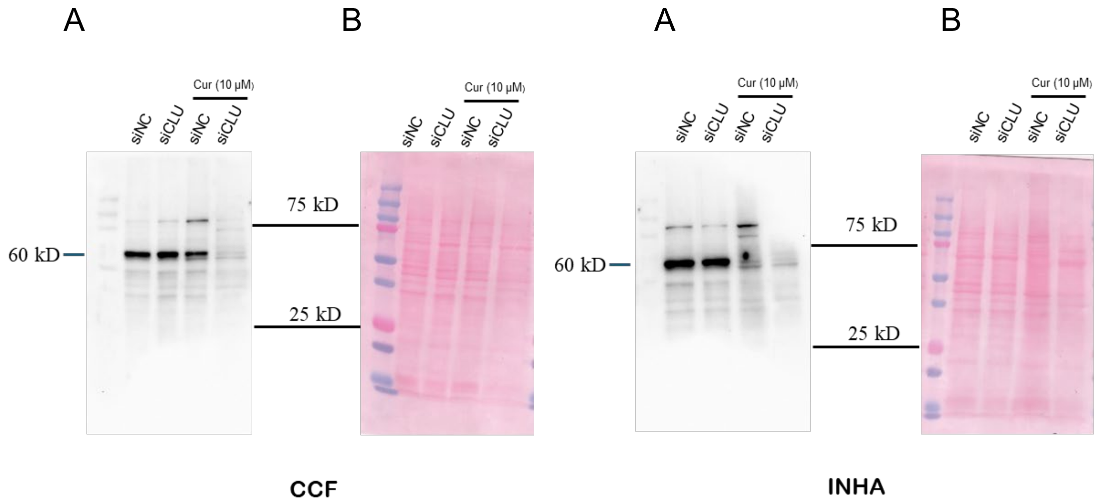
A
B
A
B
Cur (10 μM)
siNC
siCLU
siNC
siCLU
60 kD
75 kD
25 kD
CCF
INHA

Bcl-xL

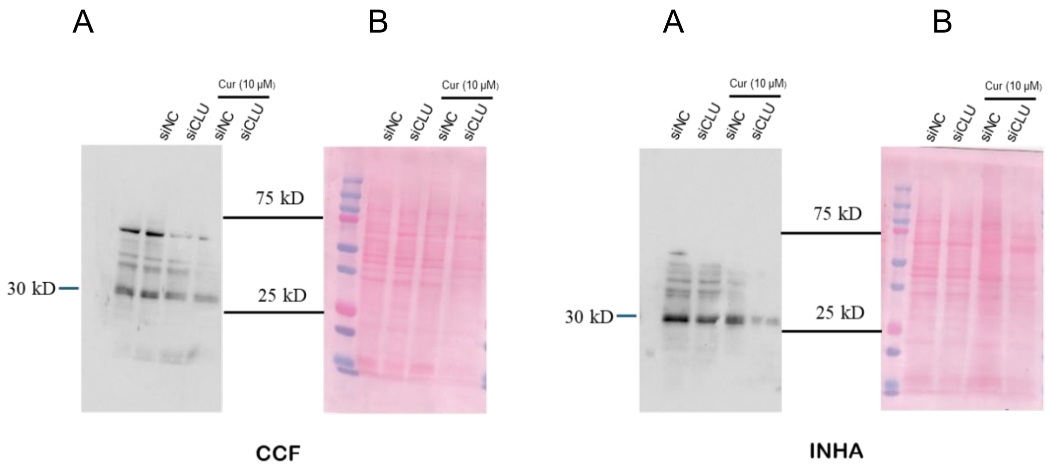
A
B
A
B
Cur (10 μM)
siNC
siCLU
siNC
siCLU
75 kD
25 kD
30 kD
CCF
INHA

Bax

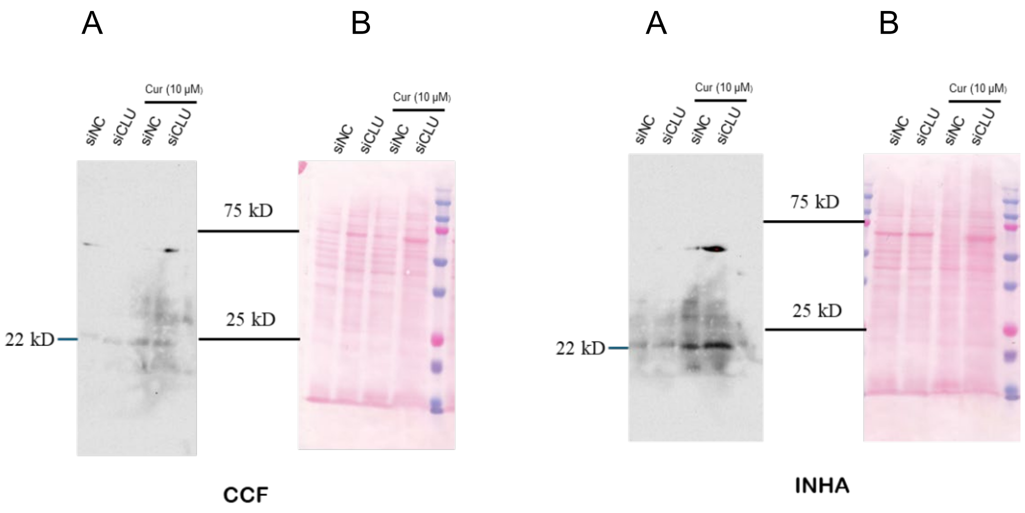
A
B
A
B
Cur (10 μM)
siNC
siCLU
siNC
siCLU
75 kD
25 kD
22 kD
CCF
INHA

Caspase 8

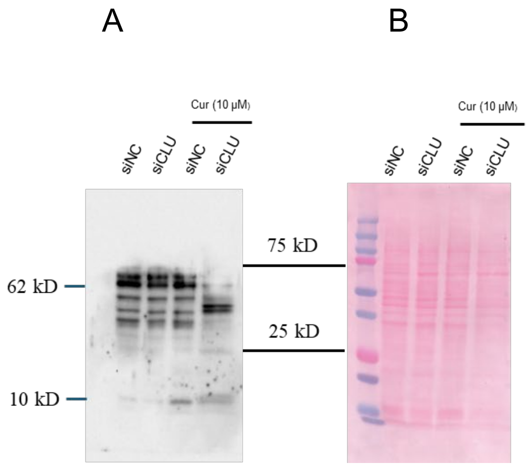

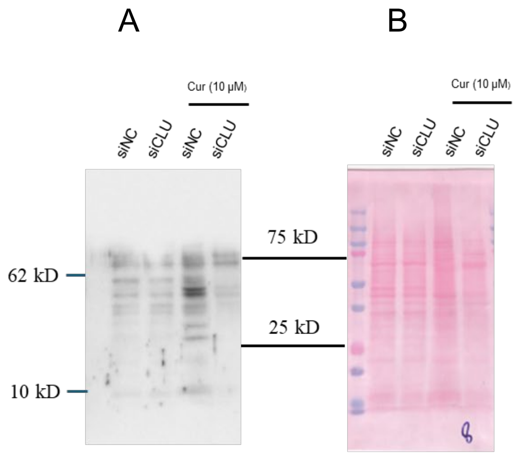

Caspase 3

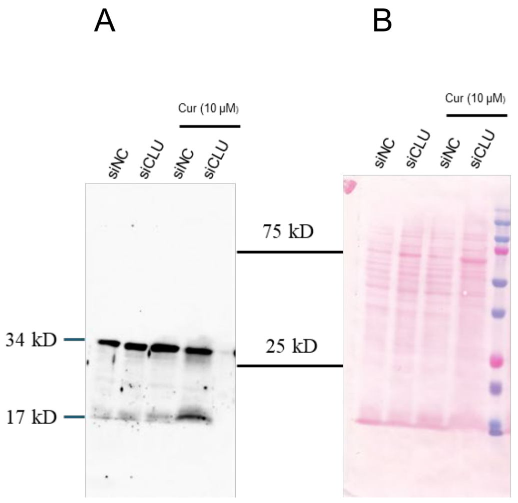

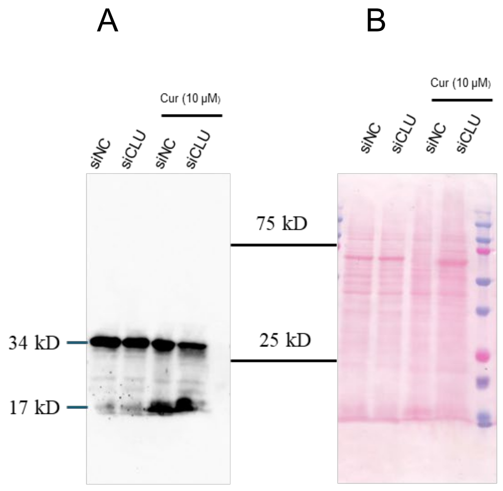

PARP

A B

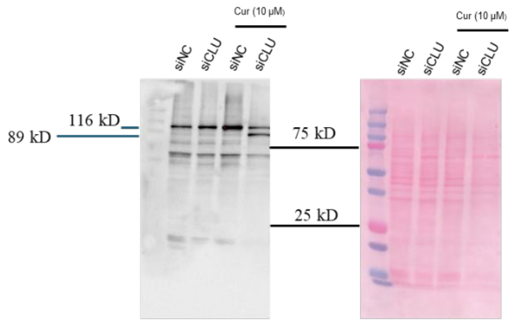

CCF

A B

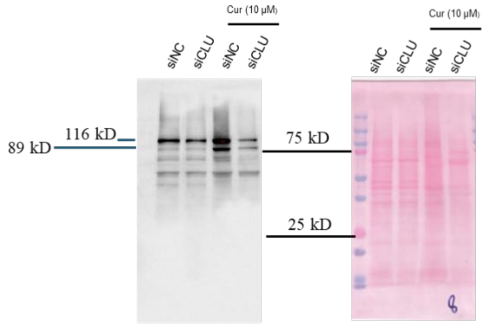

INHA
